# Evolutionary dynamics of a conserved sequence motif in the ribosomal genes of the ciliate *Paramecium*

**DOI:** 10.1186/1471-2148-10-129

**Published:** 2010-05-04

**Authors:** Francesco Catania, Michael Lynch

**Affiliations:** 1 Department of Biology, Indiana University, 1001E 3rd Street, Bloomington, IN 47405, USA

## Abstract

**Background:**

In protozoa, the identification of preserved motifs by comparative genomics is often impeded by difficulties to generate reliable alignments for non-coding sequences. Moreover, the evolutionary dynamics of regulatory elements in 3' untranslated regions (both in protozoa and metazoa) remains a virtually unexplored issue.

**Results:**

By screening *Paramecium tetraurelia*'s 3' untranslated regions for *8-mers *that were previously found to be preserved in mammalian 3' UTRs, we detect and characterize a motif that is distinctly conserved in the ribosomal genes of this ciliate. The motif appears to be conserved across *Paramecium aurelia *species but is absent from the ribosomal genes of four additional non-*Paramecium *species surveyed, including another ciliate, *Tetrahymena thermophila*. Motif-free ribosomal genes retain fewer paralogs in the genome and appear to be lost more rapidly relative to motif-containing genes. Features associated with the discovered preserved motif are consistent with this *8-mer *playing a role in post-transcriptional regulation.

**Conclusions:**

Our observations 1) shed light on the evolution of a putative regulatory motif across large phylogenetic distances; 2) are expected to facilitate the understanding of the modulation of ribosomal genes expression in *Paramecium*; and 3) reveal a largely unexplored--and presumably not restricted to *Paramecium*--association between the presence/absence of a DNA motif and the evolutionary fate of its host genes.

## Background

Conserved motifs in 3' untranslated regions (UTRs) have been identified in several organisms, including vertebrates [1,2], *Drosophila *[3-6], nematodes [6,7], *S. cerevisiae *[8], plants [9] and protozoa [10,11]. The function of these preserved sequences has often been revealed and may vary, being for example associated with cytoplasmic localization of the motif-containing mRNA and/or modulation of gene expression level. The regulatory aspect of conserved 3' UTR sequences has received large attention in recent years, and it has become apparent that 3' UTRs motifs often play a major role in post-transcriptional regulation [12], via their binding with microRNAs (miRNAs) [2,13]. Despite these crucial functions and some significant efforts to catalogue 3' UTR signals [2,8], we still know relatively little of the evolutionary dynamics of these *cis*-regulatory sequences.

In this article, we report the discovery of a conserved motif in the 3' UTRs of genes in the ciliate *Paramecium tetraurelia *and describe a number of features associated with this sequence (e.g., presence/absence across non-*Paramecium *species, evolutionary fate of the genes that carry the motif in their 3' UTR, and putative association with miRNAs). The detected motif--GUACAUUA--and a number of its variants are also conserved in mammalian 3' UTRs [2]. However, while no particular association with any gene class is reported for mammals, in *P. tetraurelia *both GUACAUUA and several of its degenerate variants are most frequently contained in the 3' UTRs of ribosomal protein genes.

## Results

### Previously described mammalian 3' UTR motifs are found in the 3' UTRs of *P. tetraurelia *ribosomal protein genes

We surveyed *P. tetraurelia *3' UTRs for the presence of conserved motifs. To this end, we did not take advantage of a motif-discovery computer program, instead we initially screened the untranslated regions for a set of *8-mers *that have been found to be conserved in mammalian 3' UTRs [2]. We found that 384 (71.1%) of these motifs hit the set of the *Paramecium *3' UTR sequences, with the vast majority of the *8-mers *yielding only one or a few hits (81% yielded ≤ 10 hits). Among the motifs that most frequently hit the *Paramecium *3' UTRs (Table 1), several share sequence similarity and partially overlap with the motif GUACAUUA, which yielded the highest number of hits, being detected in 127 3' UTRs.

**Table 1:** The number of hit 3' UTRs and the most frequent common function of the host genes are shown for the each of the most recurrent sequence motifs.

	Sequence motif	Observed hits	Expected hits	Protein function*
**1**	GUACAUUA	127	13 (3.67)	Ribosomal (88.5%)
**2**	UGUACAUU	47	11 (3.12)	Ribosomal (61.5%)
**3**	ACAAUCAU	36	15 (3.52)	-
**4**	UAUGCAAA	35	12 (3.82)	-
**5**	UUAUGCAA	34	12 (4.25)	-
**6**	AUGUACAU	33	12 (3.30)	Ribosomal (72.4%)
**7**	UUUAUGCA	32	12 (3.65)	-
**8**	AUAUGCAA	30	14 (4.21)	-
**9**	UGUACAAU	30	12 (3.16)	Ribosomal (65.2%)
**10**	GUACAUUU	29	12 (3.90)	Ribosomal (77.8%)
**11**	UUGCAAUA	29	13 (3.37)	-
**12**	UAUGCAAU	25	13 (3.25)	-
**13**	UAUGUACA	25	13 (3.49)	Ribosomal (61.1%)

Sequences that overlap the most abundant motif (GUACAUUA) are underlined. The expected average number of hits (standard deviation is in parentheses) is obtained by screening 25 sets of randomly generated DNA sequences, whose length and nucleotide composition are identical tothe 3' UTRs sequences ofthe original dataset.  * = estimate based on genes with annotated function

The analysis of the function of the genes containing the most recurrent motifs revealed a pronounced association with ribosomal genes (Table 1). 100 out of 113 genes with annotated functions that contain the sequence GUACAUUA code for ribosomal protein-coding genes (the motif is additionally found in the 3' UTRs of two likely ribosomal genes that are annotated as part of putative chimeric transcription units in the *P. tetraurelia *genome, *GSPATP00006003001 *and *GSPATP00004104001*). In these genes, the start of the GUACAUUA motif is positioned at an average distance of 18.95 bp (SD = 5.12) from the translation termination codon. The function of the 11 remaining genes is provided in Table 2; most of these genes code for proteins that are membrane-associated or involved in membrane biogenesis, and for factors that participate in transcription, RNA processing and regulation, and translation.

**Table 2:** List of *P. tetraurelia *non -ribosomal protein-coding genes that contain the 3' UTR motif GUACAUUA.

GENE MODEL	MOLECULAR FUNCTION	BLAST score	BLAST E-value
GSPATP00003019001	Hypothetical protein	60	7e-009
GSPATP00005027001	Eukaryotic translation initiation factor	123	7e-028
GSPATP00016207001	Guanine nucleotide-binding protein	229	8e-060
GSPATP00039830001	Asparagine synthetase	112	7e-025
GSPATP00000284001	Asparagine synthetase	263	1e-069
GSPATP00018093001	AMP-binding enzyme	337	8e-092
GSPATP00015762001	Membrane transporter	97	1e-019
GSPATP00033344001	DNA-directed RNA polymerase I	980	0.0
GSPATP00026659001	RNA-binding (PUF) protein	105	1e-022
GSPATP00031576001	Phosphatidylserine decarboxylase	162	1e-039
GSPATP00005327001	Nucleolar protein NOP58	337	4e-092

**Figure 1:**
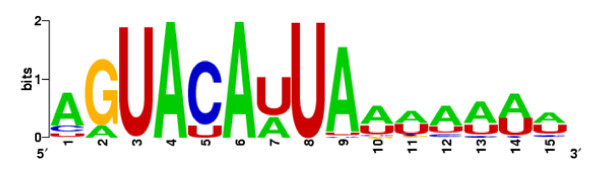
**Nucleotide composition of the common *15-mer *motif detected in the 3' UTRs of ribosomal protein genes in *P. tetraurelia***. The core region (positions 2:9) reflects the highly preserved 3' UTR mammalian motif.

We collected all genes that code for ribosome-associated proteins in *P. tetraurelia *(n = 472, on the basis of BLAST best hits against the UNIPROT database), and used the program MEME to screen their 3' UTRs, to verify both that the most likely width of the conserved motif in the ciliate is eight bases as in mammals and that these 3' UTRs contain no common motifs other than those we detected. The motif-discovering software found a single, highly conserved (E-value = 3.3e-331) motif of 15 nucleotides, which clearly contains a major *8-mer *core, essentially reflecting the previously described mammalian motif we found in the initial screening (Figure 1).

We next investigated the number of ribosomal genes containing single-nucleotide degenerate variants of the motif GUACAUUA and found that 224 (out of 472) of the 3' UTRs show a single mismatch to the motif. The average distance of the set of GUACAUUA single-nucleotide variants from the translation termination codon, as calculated for 194 unique hits, is 17.49 bp (SD = 13.57) but decreases to 16.32 bp (SD = 6.86) when two outliers (105 bp and 154 bp distant from the termination codon) are not accounted for in the analysis.

We noticed that only fifteen of the twenty-four possible single-nucleotide degenerate GUACAUUA variants were contained in the ribosomal 3' UTRs. In particular, we consistently detected 1) no motif variant with a base other than adenine at the third and fifth position, 2) no motif variant with either cytosine or guanine at the sixth position, and 3) a low frequency of adenine and cytosine as well as the absence of guanine at the seventh motif position (Figure 1).

We used the observations above, i.e., which nucleotides are either fixed or show highest frequency at each of the eight motif sites, to screen ribosomal 3' UTRs that contain neither GUACAUUA nor any of its single-degenerate variants, for a number of further degenerated, yet putatively functional, GUACAUUA variants. Specifically, we used the following degenerate motif (AGU)**UA**(CU)**A**(AU)**U**(AU), where the most degenerate nucleotides are presented within brackets, while the fixed nucleotides are in bold. Henceforth, we will refer to this set of motif variants using the consensus sequence DUAYAWUW, according to the appropriate IUPAC annotation. The consensus motif is positioned at a distance of 16.79 bp (SD = 11.37) from the translation termination codon. Sixty-two ribosomal genes (out of 472) did not contain the DUAYAWUW motif in their 3' UTR (Additional file 1).

### GUACAUUA and its variants appear conserved in *Paramecium *but not in other species

We examined the degree of conservation of the detected ribosomal 3' UTR motif for four genes across multiple *Paramecium *species and verified that the ribosomal motif is conserved at this level--the inter-specific degree of sequence divergence was relatively low across the whole 3' UTRs however (data not shown). To further characterize the motif conservation across *Paramecium *species, we then BLASTed the motif-containing *P. tetraurelia *ribosomal 3' UTRs against the unassembled contigs of the newly sequenced (yet unreleased) *Paramecium biaurelia *macronuclear DNA. The net average pairwise divergence at nuclear silent sites between *P. biaurelia *and *P. tetraurelia *ranges between 0.30 and 0.45 (depending on the inclusion of outlier strains [14]). The analysis of the putative orthologous 3' UTRs in *P. biaurelia *suggests that the exact motif GUACAUUA is also conserved in this species. Specifically, when we BLAST the 100 GUACAUUA-containing ribosomal 3' UTRs against the *P. biaurelia *macronuclear DNA, we retrieve hits for 88 of them and in the 47 cases for which we could infer (putative) orthology, the motif was virtually always completely conserved (46 out of 47)(Additional file 1).

Finally, we screened the ribosomal 3' UTRs of four additional species, *T. thermophila*, *H. sapiens*, *D. melanogaster *and *A. thaliana *and found that none of these species contains an excess of the ribosomal motif conserved in *P. tetraurelia *(Table 3). For each of these four species, we further verified whether the motif GUACAUUA significantly hits 3' UTR sequences of gene classes that are not ribosomal. Gene Ontology annotation tools, g:Profiler [15] and GOEAST [16], and visual inspection hint at a slight enrichment for this motif in genes that code for proteins that are involved in: in *H. sapiens*, regulation of macromolecule biosynthesis and amino acid transport across the plasma membrane (respectively 67 and 5 proteins out of a total of 273 proteins); in *D. melanogaster*, cell projection organization (12 out of a total of 117 proteins); in *A. thaliana*, binding (32 out of a total of 92 proteins). No clear enrichment in a particular gene classis observed for *T. thermophila*.

**Table 3:** The number of occurrences of the GUACAUUA and its single nucleotide degenerate variants is examined in *Paramecium *and four additional species (*T. thermophila*, *D. melanogaster*, *A. thaliana *and *H. sapiens*).

Species	Number of ribosomal 3'UTRs	GUACAUUA (count)	GUACAUUA single nucleotide degenerate variants (count)†
***P. tetraurelia***	472	100 (1)	246 (38)
***T. thermophila***	81	1 (1)	16 (35)
***D. melanogaster***	186	1 (2)	19 (37)
***A. thaliana***	432	2 (3)	28 (77)
***H. sapiens***	283	1 (3)	43 (83)

A motif is considered over- represented if its frequency in the set of ribosomal 3' UTRs is significantly higher (P < 0.01) than its frequency in the set of non-ribosomal 3' UTRs. Average numbers of hits expected by chance arein parentheses.  † = multiple hits are included

### Ribosomal genes that lack the motif or retain only uncommon 3' UTR motif variants are lost more rapidly

The DUAYAWUW motif may serve a significant role for the biological activity of ribosomal genes in *P. tetraurelia*. Thus, we hypothesized that the protein-coding sequence of ribosomal genes that do not contain the 3' UTR DUAYAWUW motif may tend to show lower levels of evolutionary constraints compared to motif-containing ribosomal genes.

**Figure 2:**
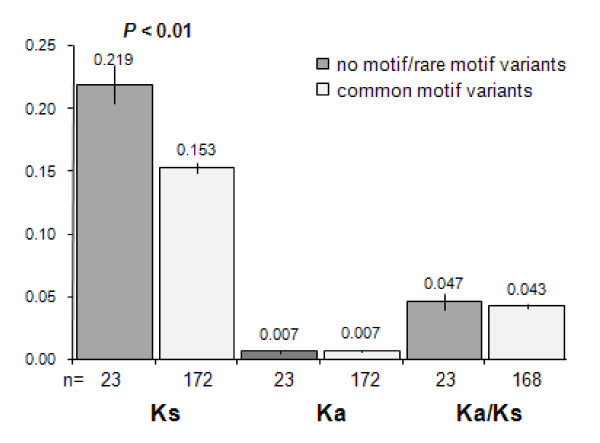
**Average *K*_*s*_, *K*_*a *_and *K*_*a*_/K_*s *_values (and standard errors) estimated for pairs of duplicated ribosomal genes (as products of the most recent polyploidization event in *P. tetraurelia*)**. Average diversity values are examined between gene pairs where both copies contain the DUAYAWUW motif and gene pairs where at least one of the copies does not contain this motif.

We tested this hypothesis by taking advantage of the most recent whole genome duplications detected in *P. tetraurelia*. This duplication event is thought to have occurred in *P. aurelia *before the emergence of the 15 species--one of which is *P. tetraurelia*--that today belong to the *P. aurelia *complex [17,18]. We found that the *K*_a_/K_s _ratio is comparable between motif-free and motif-containing genes. However, synonymous sites, but not non-synonymous sites, appear to evolve more rapidly between paralogous genes where at least one of the two genes lacks the 3' UTR DUAYAWUW motif (*t-test*, P_(1-tail) _= 0.0075) (Figure 2), and this faster sequence evolution extends to the 3' UTR sequences (*U-test*, P_(1-tail) _= 0.0365). We also observed that in paralogous genes containing DUAYAWUW, the motif sequence is highly conserved (i.e., the motif sequence is typically identical between paralogs) and that no correlation exists between motif diversity and *K*_*s *_(data not shown). Also, motif-containing ribosomal genes appear to retain a larger number of paralogs and to be more highly expressed compared to genes that only contain the rarest motif variants or no motif at all (5.08 *vs *4.00, M-W test, *P*_(1-tail) _= 0.0014; average ESTs number: 15.33 vs. 9.60, M-W test, *P*_(1-tail) _< 0.001). Finally, the set of ribosomal genes that do not have a paralog that derives from the recent polyploidization event (n = 28) is enriched with motif-free genes. Specifically, the latter set contains 11 (of 47) motif-free genes and only 17 (of 373) motif-containing genes (Fisher's Exact Test, *P*_(1-tail) _= 2.5 × 10^-4^).

### Can the detected conserved motif be a miRNA target?

The conserved mammalian 3' UTRs motifs described by Xie et al. [2] show a distinct bias in DNA strand location, being preferentially conserved in the coding strand. This observation, jointly with both the 8-base motif length and the high frequency of an adenine as the ending nucleotide, led these authors to hypothesize a regulation activity associated with miRNAs, a hypothesis subsequently confirmed by experiments for a number of these motifs.

When we studied the strand specificity of the ribosomal motif (and motif variants), we found a pronounced abundance of the motif on the coding strand (it must be noted that a putative equally functional complementary motif is clearly just as abundant on the opposite strand). Indeed, the frequencies we reported above only refer to the presence of both GUACAUUA and its variants in the forward strand. When we searched for this motif on the complementary strand, we no longer observed an overrepresentation, and in fact we found no hits for the motif GUACAUUA on the complementary strand. The strand specificity we observe suggests that this 3' UTR motif acts at the RNA rather than at the DNA level and thus plays a role in post -transcriptional regulation.

How likely is it that the newly discovered motif is also a miRNA target in *Paramecium*? We addressed this question by taking advantage of some of the findings of a recent large-scale study of miRNAs in metazoans [19]: 1) uracil (U) is the most frequent nucleotide in mature miRNA sequences--being particularly enriched at the first and the ninth nucleotide positions, i.e., sites that immediately flank the miRNA "seed" region, which is believed to have a critical role in binding the target sequences; and 2) guanine(G) is significantly depleted at position one.

Assuming that protozoans share the same or similar features with miRNAs in metazoans, an initial inspection of the sequence that is reverse complementary to the conserved ribosomal motif, i.e., UAAUGUAC (this sequence represents a portion of the putative miRNA sequence and includes, underlined, the seed region), is broadly consistent with features that are reminiscent of miRNA in metazoans (i.e., U is enriched at the first position, where G is scarcely found). A closer analysis of the motif profile points to a higher level of resemblance. Specifically, the pictogram representation of the consensus motif presented in Figure 1 reveals the frequent occurrence of an A (or less frequently a C or a U, but very rarely a G) upstream of the consensus ribosomal motif, at position nine; such an enrichment in As is illustrated by the high frequency (76%) with which an A immediately precedes the 100 GUACAUUA motifs that we found in the ribosomal 3' UTRs of *P. tetraurelia *(the frequency of adenines in the examined set of 3' UTRs is 44.1%). This suggests that the putative complementary miRNA sequence is likely to contain a U not only at position one but also at position nine, in agreement with what has been described for miRNA in metazoans.

If the complementary UAAUGUAC sequence is part of the miRNA sequence that targets the conserved ribosomal motif, then one could expect to find a region of the macronuclear DNA of *P. tetraurelia *containing this motif (along with its complementary version) and having a stable secondary structure that is typical of miRNA precursors. Alternatively the putative miRNA sequence could be located in the micronuclear DNA--in *Paramecium *there are two nuclei, the macronucleus (the somatic nucleus) and the micronucleus (the germline nucleus). In an attempt to identify a putative pre-miRNA, we screened the available *P. tetraurelia *macronuclear genome [20] for stem-loop structures containing the motif seed and its complementary version that are separated by up to 200 base pairs and flanked upstream and downstream by an arbitrary number of bases (10, 20 and 30 bps).

**Figure 3:**
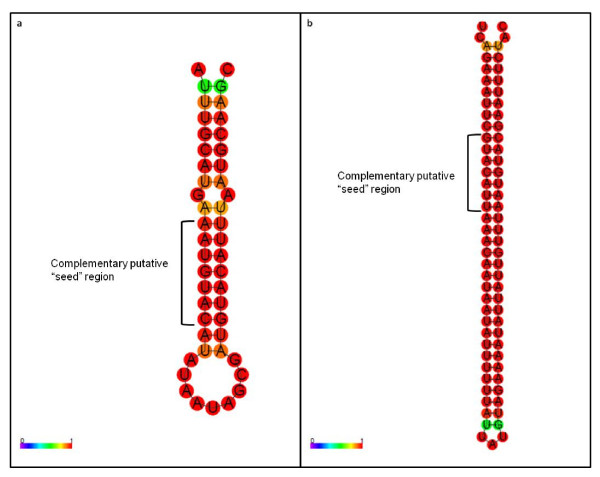
**Structure of the two candidate precursor miRNAs in *P. tetraurelia***. a) candidate pre-miRNA (location in macronuclear genome: scaffold_567:869-915); b) candidate pre-miRNA (this is a portion of an EST [cDNA clone LK0ADA28YP05; collected at conjugation (beginning of meiosis)] that can be only partially mapped to the 3' end region of three 60S ribosomal protein-coding gene L1 that are located in scaffold_161: 34738-35490; scaffold_253: 730-1589; and scaffold_151: 51790-52544). Color scale displays base-pairs probability.

This screening yielded a total of 284 candidate pre-miRNAs. For each of these candidate elements we assessed the stability of the secondary structure by measuring: 1) the minimum free energies (MFE), as predicted by the *RNAfold *program [21]; the parameter AMFE ([MFE/length]*100)), which provides an estimate of stability that is not influenced by differences in RNA sequences length; and MFEI (AMFE/(G+C)%), an index that appears to be valuable in distinguishing miRNAs from other coding and non-coding RNAs [22], and whose absolute values are consistently close or higher than 0.85 when experimentally confirmed miRNAs in metazoans are examined [19,22]. The study of these parameters across the candidate pre-miRNAs led to the identification of a putative pre-miRNA that, irrespective of the arbitrary number of flanking bases, showed consistently both low AMFE values (-28.69 ± 7.25), which are comparable to those detected for confirmed miRNAs in metazoans [19], and absolute values of MFEI close to or higher than 0.85 (0.98 ± 0.20). This candidate pre-miRNA, whose MFE structure is shown in Figure 3a, matches none of the currently available *P. tetraurelia *ESTs, has a single hit in the *P. tetraurelia *genome and is located in a region that is devoid of genes.

It is worth noting that a screening of the *P. tetraurelia *EST sequences led to the identification of an additional candidate pre-miRNA. This further putative pre-miRNA is detected in an EST that only partly matches the 3' end of a 60S ribosomal protein-coding gene. Specifically, while the 5' end sequence of this EST matches the ribosomal gene sequence as well as the homologous region of other ESTs that have been mapped to this gene, its 3' end sequence shows a unique profile that differs both from the sequence of the genomic DNA and the remaining ESTs that match this region. This EST's peculiar 3' end sequence is capable to form an extremely stable structure (AMFE average: -43.61 ± 9.26; MFEI average: -2.47 ± 0.36) (Figure 3b).

### Additional motifs in the 3' UTRs of *P. tetraurelia*

We finally screened the *P. tetraurelia *3' UTRs for the presence of the hexanucleotide typically associated with polyadenylation in mammals (AAUAAA), and *k-mer *motifs that were previously found to be highly conserved in worms (n = 442) and flies (n = 497) [6]. The latter two sets of motifs include both verified and putative miRNA targets as well as GU and AU-rich elements and PUF-binding sites. We found that the AAUAAA motif hits the whole set of *P. tetraurelia *3' UTRs 1780 times (the number expected by chance--obtained by screening 25 sets of randomly generated DNA sequences, whose length and nucleotide composition are identical to the 3' UTR sequences of the original dataset--is 1599 (SD = 27.81)), and is located an average distance of 29.39 bp (SD = 30.92) from the translation termination codon. We also found that several U-rich *k-mers *that are conserved in worm and/or flies, appear to be overrepresented in *P. tetraurelia *3' UTRs. Aside from the GUACAAU and UGUACAUU motifs--which resemble the GUACAUUA sequence we describe above, and as such appear to be preferentially located in ribosomal 3' UTRs (64.5% and 43.3% respectively)--these overrepresented motifs are not clearly associated with a specific class of proteins, occur at various frequencies, and are located at different positions along the 3' UTRs (Table 4).

**Table 4:** Highly conserved motifs in worms and/or flies [6] detected in *P. tetraurelia *3' UTRs.

Motif	Observed hits	Expected hits	Distance (bp) from translation termination codon
UAAAUAAAU	165	121 (0.97)	26.46 (37.08)
UAUAUAUA	689	246 (3.56)	23.52 (25.23)
UGCAUUU	146	64 (1.76)	35.46 (44.36)
UGUGUAU	106	53 (0.99)	26.98 (32.07)
UUUUUAUA	175	284 (2.14)	28.77 (40.40)
UGUACAUU	47	11 (1)	21.83 (20.20)
GUACAAU	109	33 (2.46)	21.55 (20.10)
UCAAUAAA	107	68 (1.27)	29.94 (28.01)
UACUAAC	12	35 (0.81)	32.33 (19.81)
UUGCAUA	130	61 (2.59)	28.75 (41.93)

The list includes the top 10 *k-mers *for which the frequency of occurrence in *P. tetraurelia *3' UTRs most deviate from the corresponding frequency expected by chance. Values in parentheses are standard deviations.

## Discussion and Conclusions

A growing body of literature is providing critical information about innovative strategies for the identification of conserved *cis*-regulatory motifs [23-25] and about the regulatory interactions between conserved UTR sequences and miRNAs [2,6,12,13,26-30]. However, the evolutionary dynamics of conserved UTR motifs remain virtually unexplored.

In this article, we have reported the discovery and the characterization of a conserved *8-mer *motif--GUACAUUA--as well as several of this motif's degenerated variants, in the 3' UTRs of *P. tetraurelia *ribosomal-protein genes. By studying the distribution frequency of GUACAUUA single-nucleotide degenerated variants, we yielded the profile of the conserved consensus motif (DUAYAWUW), where four nucleotides are perfectly conserved and nucleotide frequencies at the remaining four positions tend to be skewed toward a single nucleotide (Figure 1). The region occupied by the preserved ribosomal sequence has overall a narrow size and the motif is relatively close to the translation termination codon (average distance of the start of the motif from termination codon is 17.9 bp [SD = 7.30]). Both the positional range and the relative distance from the termination codon are smaller when compared to the corresponding values estimated for additional motifs we found to be overrepresented in *P. tetraurelia*3' UTRs (Table 4).

The study of the degree of conservation of the detected motif across *Paramecium *and non-*Paramecium *species shows that this motif is broadly conserved across multiple *Paramecium *species but is typically absent from the ribosomal genes of three multicellular organisms and, most notably, from another ciliate species (*T. thermophila*), which is distinct both morphologically and molecularly [31], yet an Oligohymenophoran like *Paramecium*. Unfortunately, the absence of genome sequence and EST information for species that are phylogenetically closer to *Paramecium *currently complicates the attempt to further trace back the evolutionary origin of the motif (but see below).

The hypothesis that the mere presence/lack of the motif can in some way influence (or be influenced by) the rate of protein-coding sequence evolution is not supported by the similar levels of constraints at non-synonymous sites observed for motif-free and motif-containing ribosomal genes (Figure 2). However, the study of *K*_s _and 3' UTR sequence variation between duplicated ribosomal genes reveals higher rates of nucleotide substitutions at silent and 3' UTR sites in motif-free ribosomal genes compared with motif-containing genes. An explanation for the association between the lack of motif and the faster evolution at silent and 3' UTR sites may be non-biological. Specifically, the motif-free ribosomal genes we used to estimate nucleotide variation may not all be the most recent paralogs, as initially assumed. A number of gene duplicates in the set of motif-free genes could result from independent (and temporally distant) events of WGD, but be considered as most recent copies if their true closely related copies have gone lost. Consistent with this hypothesis, we find that motif-free ribosomal genes retain significantly fewer paralogs and are lost more often relative to motif-containing genes. Also, *K*_s _estimates between motif-free gene copies tend to be high (>0.25) when only two duplicates are detected in the genome, a condition that may more easily lead to classify incorrectly the gene copies as recent duplicates. Finally, as the identification of the WGD paralogs involved also the study of the synteny between blocks of duplicated genes [20], by visual inspection we find that similar features can be shared between motif-free ribosomal duplicates and their flanking genes, i.e., flanking genes retain also only one paralog and/or show comparable high levels of divergence at silent sites (data not shown). The latter observation implies that the hypothetical incorrect assignment of recent paralogy could not only involve the motif-free ribosomal genes but extend also to their flanking genes.

Finally, the observation that the GUACAUUA sequence is typically located on the forward DNA strand is consistent with the idea that this motif is involved in post-transcriptional regulation--although the alternative explanation that the motif is a DNA binding protein motif cannot be ruled out for now. Three observations hint at the possibility that the newly described motif plays a role in modulating expression of the host gene. First, motif-containing and motif-free genes are differentially expressed, with motif-containing genes being more highly expressed compared to motif-free genes, a finding that along with the higher levels of retention described for motif-containing genes is reminiscent of the reported positive correlation between gene retention and gene expression in *Paramecium *[32]. Second, the GUACAUUA sequence is reminiscent of the PUF-binding site (UGUAnAUA), and PUF proteins--a family of mRNA-binding proteins--are known to repress gene expression, either by inhibiting the translation or by enhancing the decay of target mRNAs [33,34]. Third, the GUACAUUA sequence closely resembles a conserved 3' UTR motif in yeast, UGUAUAUUA, that mediates the destabilization of the host mRNA [8]. Intriguingly, this yeast motif is also enriched in ribosomal genes, and has a mammalian counterpart that is the target of a miRNA, miR-381 [8]. The implications of the latter observations are twofold: 1) the GUACAUUA motif was probably not gained independently in mammals and *Paramecium *but emerged in the common ancestor of the surveyed species and underwent either secondary loss or switches in expressed genes; the GUACAUUA motif may too be a binding site for a miRNA, which would be expected to have co-evolved with the core motif. While an experimental validation is clearly needed to provide any solid support for the latter hypothesis, the possibility of a connection between the motif and miRNAs is twofold intriguing: 1) the putatively regulated genes (i.e., ribosomal protein-coding genes) would not be lowly or only moderately expressed genes, as genes that are commonly thought to be typical targets of miRNAs, and 2) aside from small RNAs that are involved in the definition of the new macronucleus [35], and a class of short RNAs that are involved in post-transcriptional gene-silencing [36], miRNAs have never been described in ciliates.

## Methods

### Motif characterization

We investigated the presence of conserved signal sequences in the 3' untranslated regions of *Paramecium*, by extracting 7647 annotated 3' UTR sequences from the *P. tetraurelia *genome database [37], and screening these sequences for motifs that have been already catalogued for other species (a flow chart is provided in Additional file 2). In particular, we used 540 *8-mers *that were previously identified in mammalian 3' UTRs [2], as well as 442 and 497 *k-mers *that are highly conserved in worms and flies respectively [6]. The procedure of gene, and thus of UTR, annotation in *P. tetraurelia *is described in Aury et al. [20] and takes advantage of cDNA libraries that include gene transcripts detected at six different physiological conditions/developmental stages. The average length calculated for the studied 3' UTRs is 52.1 bp (CV = 0.68).

As conserved signals may be more likely to be shared among genes coding for proteins that have similar functions, we characterized the molecular role of *P. tetraurelia *genes having an annotated 3' UTR, by BLASTing the corresponding protein sequences against the UNIPROT database [38]. We used an E-value cut-off of 10^-7 ^and gap opening and gap extension penalties equal to 10 and 1 respectively. We assigned a given molecular function to each of the examined *P. tetraurelia *proteins, according to the information drawn from the BLAST first best hit.

We used the motif discovery MEME software [39] to verify the exclusive presence and the most likely width of the conserved motif we discovered in genes coding for ribosomal proteins (see below) and Weblogo [40] to produce a graphical view of the degree of conservation at every site.

### Interspecific conservation of the motif

We performed PCR and DNA sequencing to assess the level of conservation of the detected *P. tetraurelia *ribosomal motif across multiple species of the *P. aurelia *complex and a species closely related to the *P. aurelia *species complex, *P. caudatum*. We designed PCR primers using the coding regions of two adjacent genes (the upstream of which being a ribosomal protein gene) and obtained PCR products spanning the intervening ribosomal 3' UTR. Due to difficulties in DNA amplification across the whole set of (or most) species surveyed, the analysis across these relatively diverged species [14] produced successful results only for a limited number of 3' UTRs (n = 4) (data not shown). We calculated the level of sequence divergence (*d*) along the entire 3' UTR using the Maximum Composite Likelihood method implemented in the software MEGA 4.0 [41].

To further verify the degree of evolutionary conservation of the motif detected in *P. tetraurelia*, we next surveyed the 3' UTRs annotated for ribosomal protein genes of four non-*Paramecium *species (*Tetrahymena thermophila*, *Homo sapiens*, *Drosophila melanogaster *and *Arabidopsis thaliana*). It is worth stressing that the *8-mer *we discovered in the *P. tetraurelia *3' UTRs, as well as the remainder of conserved mammalian motifs we used for our initial screening, had not been explicitly associated with specific gene functions in mammals. Using the same procedure described above to infer the molecular function of *P. tetraurelia *genes, we collected the following number of unique ribosomal genes (*i.e*. isoforms are not counted) for each of the additional species surveyed: 142 (*T. thermophila*), 282 (*H. sapiens*), 176 (*D. melanogaster*), 521 (*A. thaliana*). For each of the latter three sets we directly extracted the annotated 3' UTRs. In *T. thermophila*, where 3' UTRs are not annotated, putative 3' UTR sequences were retrieved by cropping 500 bp of genomic DNA, downstream of each ribosomal gene, and blasting the cropped region against the whole set of *T. thermophila *ESTs. The EST hit that extends most downstream of each of the translation termination codons was used as 3' UTR for this study.

### Levels of sequence divergence between recently duplicated ribosomal genes

To verify whether a correlation exists between the evolution of the motif sequence and that of the coding sequence of the gene that contains it, we estimated the level of sequence divergence between recently duplicated *P. tetraurelia *macronuclear ribosomal protein-coding genes (as derived from the most recent event of whole genome duplication (WGD) in *P. tetraurelia*). Specifically, we grouped separately pairs where both genes contain the discovered conserved motif and pairs where at least one of the two genes was motif-free. We verified the absence of the conserved motif in all the motif-free genes that only contained a short (*i.e*. presumably incomplete) annotated 3' UTR. In one case we found that the 9 bp annotated 3' UTR of the gene model *GSPATP00010990001 *contained the motif within the following 10 bp and we included this gene in the set of motif-containing genes.

For each of the ribosomal genes in *P. tetraurelia*, we retrieved the most recent paralog according to the 'ALL-against-ALL' Blast analysis and the analysis of the synteny between blocks of duplicated genes performed by Aury et al. [20]. We aligned the duplicate sequences with ClustalW [42], verified the existence of indels, suspicious introns, correct UTRs and gene predictions (see below), and performed manual editing if needed. We used the Kumar method implemented in the program MEGA 4.0 to estimate the average *K*_*a*_, *K*_*s *_and *K*_*a*_/K_*s*_. We used both the *t*-test and the Mann-Whitney *U-*test to assess the significance of the observed differences. We performed the *t-test *after square root transforming the raw data and testing both for normality (*K-S test*) and for equality of variances (*Levene's test*). After data transformation, a normal distribution could be obtained consistently only for the *K*_*s *_estimates.

We also estimated nucleotide diversities (corrected for multiple substitutions [43]) for all pairs of paralogous ribosomal 3' UTRs, after the removal of the *8-mer *motif. When 3' UTRs contained more than one motif copy, we randomly removed one of the copies.

### Inspection of the ribosomal genes' coding sequences

To calculate estimates of levels of sequence divergence we used alignments of putatively functional genes, i.e., we discarded pseudogenes, a condition that is not uncommon for genes that code for ribosomal proteins. The vast majority of alignments produced by ClustalW were highly reliable--no manual editing was needed, and the sequences always had virtually identical length. In nine cases (one in the motif-free set and eight in the motif-containing set), the presence of indels not verified by ESTs or a high variability, both in the very 5' end of the gene sequences, complicated the local alignment. In these cases, we only aligned the corresponding sequences starting from the first ESTs verified site when ESTs were available, or from the site where the alignment started to be unambiguous, when ESTs were not available. We include these gene pairs in our analysis and verified that their removal did not affect our conclusions.

Further, we detected a limited number of non-frame-preserving indels. Such mutations typically lead to incorrect introns, UTR or gene predictions and might reflect a pseudogenization event. We verified and rejected the occurrence of every indel (presumably deriving by sequencing errors), by examining the corresponding ESTs and re-estimated diversity after reintroducing the erroneously eliminated coding regions. When ESTs were not available, we excluded the gene pairs containing the non-frame-preserving indel(s) from the analysis, as these genes may represent pseudogenes.

Finally, in an additional attempt to detect pseudogenes in our study, we examined intronless genes. The reason for the latter analysis is that pseudogenes could arise after an event of reverse transcription followed by reinsertion into the genomic DNA (processed pseudogenes). We found only one gene with no introns. This gene, for which no ESTs are available, is a motif-containing ribosomal gene and shows a relatively high *K*_a _value (*K*_a _= 0.10) and a predicted premature translation termination codon. We removed the corresponding gene pair from our analysis.

## Authors' contributions

FC conceived and conducted the study. FC and ML wrote the manuscript.

## Supplementary Material

Additional file 1 **Lists of motif-containing and motif-free ribosomal genes**. The lists include the name of the ribosomal genes and their corresponding 3'UTR sequences with/without the GUACAUUA motif, with the single-nucleotide degenerate GUACAUUA variants and the consensus DUAYAWUW motif. The list includes also the (putative) orthologous 3'UTR sequences from *P. biaurelia*. Click here for file

Additional file 2 **Flow-chart**. Flow-chart indicating the bioinformatic procedures used in the study. Click here for file
