# Supplementary figures and images for: Evolutionary dynamics of a conserved sequence motif in the ribosomal genes of the ciliate Paramecium
Source: BMC Evol Biol. 2010 May 4;10:129. doi: 10.1186/1471-2148-10-129 (PMC2874801; doi:10.1186/1471-2148-10-129)

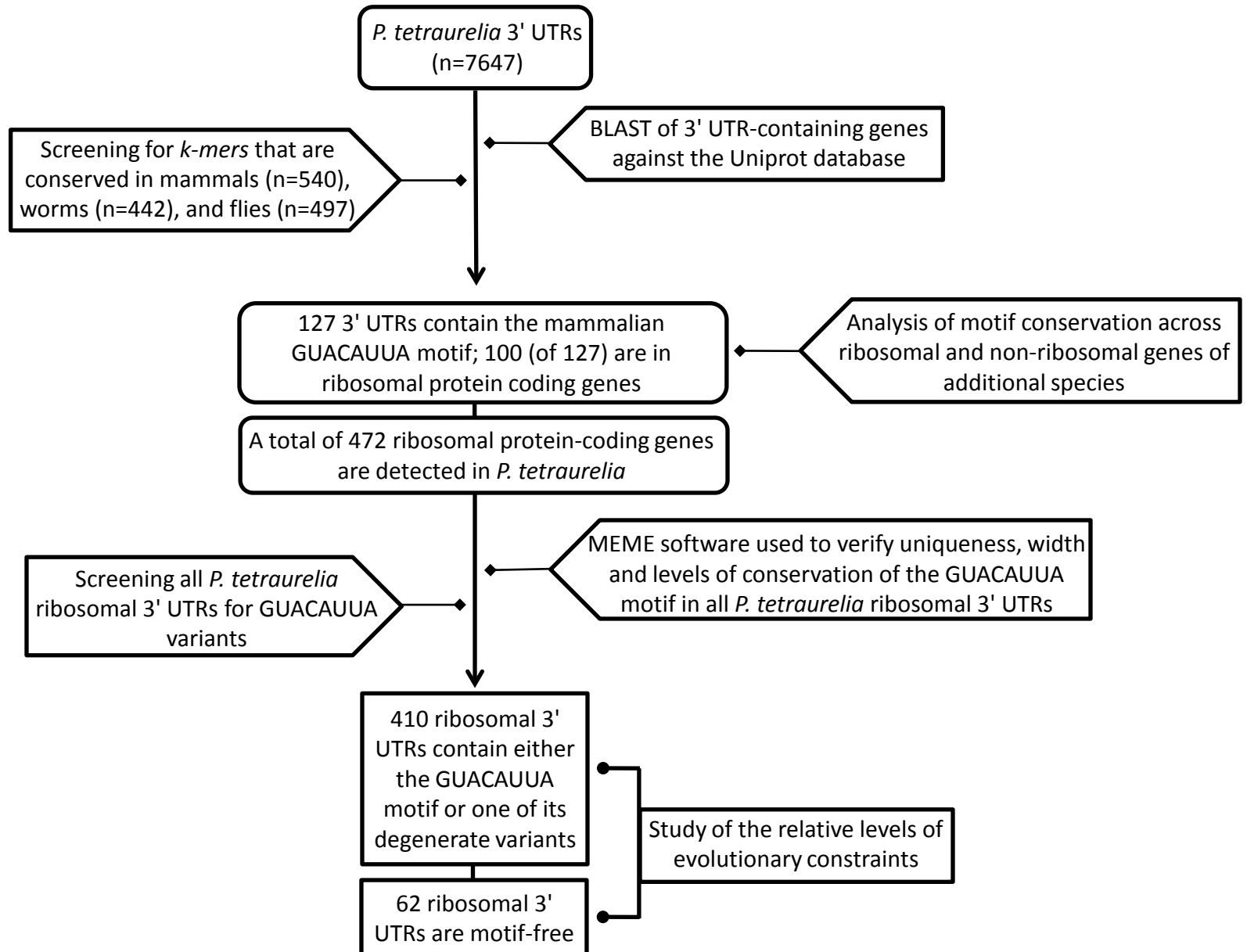

Supplement: Additional file 2 — Flow-chart. Flow-chart indicating the bioinformatic procedures used in the study. [file 1471-2148-10-129-S2.PDF]
